# Mechanistic Insights into the Photocatalytic Degradation of Chlorophenols in Aqueous Systems via Nonlinear Kinetic Modeling

**DOI:** 10.3390/toxics14060480

**Published:** 2026-05-30

**Authors:** Liliana Bobirică, Cristina Orbeci, Giovanina-Iuliana Ionică, Constantin Bobirică

**Affiliations:** Department of Analytical Chemistry and Environmental Engineering, Faculty of Chemical Engineering and Biotechnology, National University of Science and Technology POLITEHNICA Bucharest, 1–7 Polizu, 060042 Bucharest, Romania; liliana.bobirica@upb.ro (L.B.); cristina.orbeci@upb.ro (C.O.); giovanina.ionica@upb.ro (G.-I.I.)

**Keywords:** photocatalysis, chlorophenols, nonlinear kinetic modeling, TiO_2_ photocatalyst

## Abstract

Chlorophenols (CPs), such as 4-chlorophenol (4-CP) and 2,4-dichlorophenol (2,4-DCP), are persistent and toxic organic pollutants commonly found in industrial effluents. This study investigates their photocatalytic degradation using a TiO_2_-based heterogeneous catalyst under UV irradiation, in the presence of hydrogen peroxide. The degradation kinetics were analyzed using both pseudo-first order and nonlinear Langmuir–Hinshelwood (L–H) models, accounting for competitive adsorption and successive oxidation of intermediates. Gas chromatography–mass spectrometry (GC–MS) identified key intermediates, including hydroquinone, catechol, chlorocatechols, and benzoquinone. Nonlinear kinetic modeling of coupled differential equations accurately reproduced the temporal profiles of both the parent compounds and their intermediates, providing mechanistic insights into multi-step hydroxylation, dechlorination, and oxidation processes. The results demonstrate that photocatalytic oxidation effectively mineralizes chlorophenols within 500–600 min, and the developed kinetic model offers a predictive tool for optimizing photocatalytic remediation strategies for chlorinated aromatic pollutants. The novelty of this study lies in the development of a nonlinear Langmuir–Hinshelwood kinetic model integrating experimentally identified degradation intermediates, competitive adsorption phenomena, and parallel photocatalytic reaction pathways for both 4-chlorophenol and 2,4-dichlorophenol oxidation systems.

## 1. Introduction

Chlorinated phenols are persistent organic pollutants that are widely encountered in industrial effluents due to their extensive use in pesticides, disinfectants, dye manufacturing, and various other chemical processes [1,2]. These compounds are highly toxic, poorly biodegradable, and chemically stable in aqueous environments, which makes their removal challenging using conventional treatment methods such as biological degradation or chemical coagulation [3,4]. Their persistence in natural waters and industrial discharges represents significant risks to both human health and aquatic ecosystems, emphasizing the need for effective remediation strategies [5,6].

Heterogeneous photocatalytic oxidation has emerged as a particularly promising approach for the removal of chlorinated phenols and other recalcitrant organic pollutants. Semiconductor photocatalysts, most notably TiO_2_-based materials and other visible-light responsive systems, can generate electron–hole pairs under ultraviolet or visible-light irradiation, initiating reactive radical species that oxidize and dechlorinate the pollutants [7,8]. These photogenerated charge carriers produce reactive species such as hydroxyl radicals (•OH) and other reactive oxygen species, which initiate oxidation and dechlorination reactions that progressively transform the pollutants into less harmful intermediates and ultimately mineralize them to CO_2_ and H_2_O [9,10].

For the rational design and optimization of photocatalytic systems, it is essential not only to evaluate overall pollutant removal efficiency but also to understand the underlying reaction kinetics and mechanistic pathways [11,12]. Photocatalytic reactions involve complex interactions between adsorbed species, dissolved reactants, and reactive radicals generated on the catalyst surface, resulting in non-linear and multi-step kinetics. Classical kinetic frameworks developed for heterogeneous catalysis provide useful conceptual guidance for interpreting these processes. Among the most widely referenced models for describing surface-catalyzed reactions are the Langmuir–Hinshelwood (L–H), Eley–Rideal (E–R), and Mars van Krevelen (M–K) mechanisms. These frameworks account for the effects of surface adsorption, reactant availability, and catalyst activity on the observed reaction rates, forming a basis for experimental design and data analysis [13,14,15].

Despite the insights offered by classical heterogeneous reaction models, the complexity of photocatalytic oxidation, particularly in systems involving chlorinated phenols, requires a more refined interpretative framework. Non-linear kinetic modelling has been demonstrated to more accurately represent the coupled effects of substrate adsorption, oxygen activation, and radical-mediated surface reactions [16,17]. Such modelling helps identify the dominant mechanistic contributions and distinguish between parallel or competing surface-reaction regimes that cannot be resolved using simplified linear approaches. Therefore, integrating non-linear kinetic analysis into experimental studies is essential for clarifying the reaction mechanism in the photocatalytic degradation of chlorinated phenols [18,19,20,21].

In this context, the present work focuses on the nonlinear kinetic modelling of the photocatalytic degradation of two representative chlorinated phenols: 4-chlorophenol (4-CP) and 2,4-dichlorophenol (2,4-DCP). Although these compounds follow broadly similar degradation pathways, they exhibit distinct adsorption characteristics, degradation rates, and intermediate distributions, making them suitable case studies for assessing the capability of nonlinear models to resolve subtle differences in reaction kinetics and surface-mediated transformations. By combining experimental degradation data with advanced nonlinear regression techniques, this study aims to quantify the kinetic parameters governing each stage of the process and evaluate the limitations of classical linear kinetic interpretations. The resulting model framework is intended to provide a deeper mechanistic understanding of photocatalytic oxidation in systems where chemically related substrates display kinetically divergent behaviors, even when their overall degradation routes remain comparable.

## 2. Materials and Methods

### 2.1. Chemicals

All chemical reagents used in this study were of analytical grade and applied without further purification. 4-CP and 2,4-DCP were procured from Fluka Chemicals (Buchs, Switzerland). A 30% (*w*/*w*) hydrogen peroxide solution (Sigma-Aldrich, St. Louis, MO, USA) was used as the oxidizing agent in photocatalytic experiments. The pH of the solutions was controlled using 1 N sulfuric acid (Sigma-Aldrich). For COD measurements, potassium dichromate, silver sulfate, potassium hydrogen phthalate, and concentrated sulfuric acid (95–97%) were used (all from Sigma-Aldrich). Solvents and drying agents employed in the extraction of photocatalytic intermediates included diethyl ether and anhydrous sodium sulfate (Sigma-Aldrich). Distilled water was employed throughout all experimental procedures.

### 2.2. Photocatalytic Experiments

**Figure 1:**
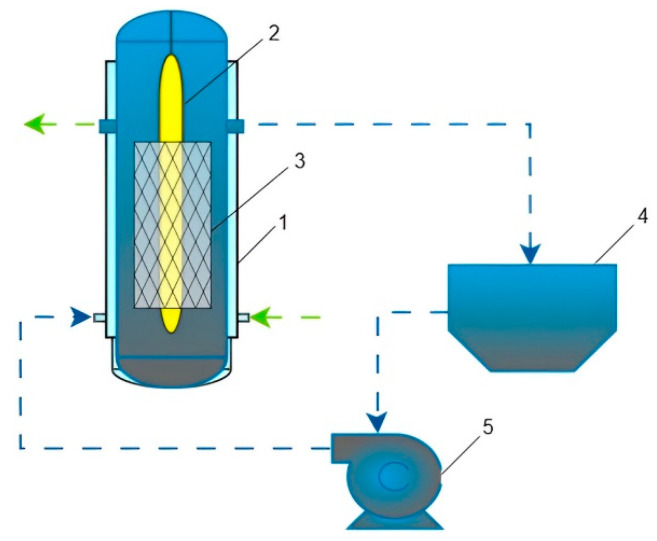
Schematic representation of the photocatalytic reactor installation: (1) reactor vessel, (2) UV lamp, (3) TiO_2_-based stainless-steel mesh support membrane, (4) feed tank, (5) recirculation pump (blue arrows indicate the flow of aqueous chlorophenol solutions and green arrows indicate the flow of cooling water).

Photocatalytic degradation was carried out in a semi-pilot-scale UV reactor (BIT srl, Milan, Italy) designed for continuous solution recirculation. An external centrifugal pump ensured steady flow of the reaction mixture during irradiation. The reactor featured a coaxial layout, integrating a UV light source, a cylindrical photocatalytic membrane (dimensions: 10 cm × 30 cm), and a water-jacket for temperature control. Figure 1 illustrates a simplified schematic of the setup.

**Figure 2:**
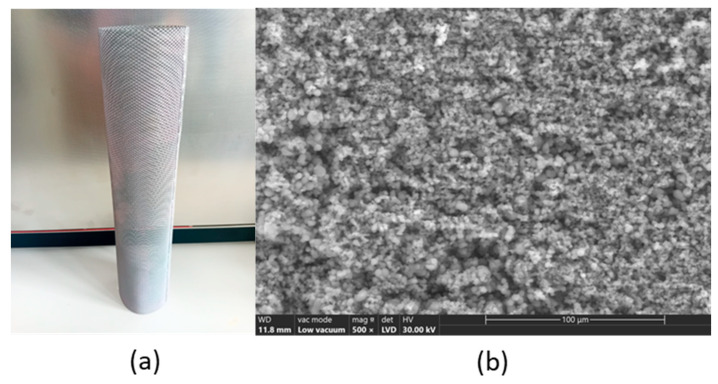
Stainless-steel/TiO_2_ membrane: (**a**) Photograph. (**b**) SEM micrograph.

The working volume of the reactor was 1.5 L, and the circulation rate was maintained at 1 L·min^−1^. A 120 W UV lamp (BIT srl, Milan, Italy) provided irradiation. The photocatalytic membrane was prepared using a cylindrical stainless-steel mesh support (10 cm × 30 cm). A TiO_2_ layer was prepared from commercial P25 powder (Degussa, Essen, Germany) and immobilized onto the stainless-steel mesh using a dip-coating procedure, replacing previously employed electrophoretic deposition methods [22]. This approach enabled the formation of a uniform and well-adhered TiO_2_ film on the metal substrate following solvent evaporation and thermal treatment (Figure 2). The low-magnification SEM image reveals a relatively uniform coverage of the stainless-steel mesh surface by TiO_2_ particles, indicating successful immobilization of the photocatalyst layer. Solutions of 4-CP or 2,4-DCP were prepared at various concentrations, expressed in mg of organic carbon per liter (mg C/L). The solution pH was adjusted to 3 prior to irradiation, based on previous optimization studies. The molar ratios of hydrogen peroxide to chlorophenols were set according to stoichiometric requirements (1:1) [22]. To further clarify the contribution of different processes to 4-CP or 2,4-DCP removal, control experiments were performed under dark adsorption, direct photolysis, and H_2_O_2_-assisted photolysis conditions for the first concentrations of both 4-CP and 2,4-DCP.

During experiments, 10 mL aliquots were periodically withdrawn to follow the degradation of organic content. Samples were filtered immediately and analyzed for COD according to the APHA 5220 D method (closed reflux, colorimetric) [23], using a Hach Lange LT 200 thermostatic reactor (Düsseldorf, Germany) and a Hach Lange DR 3800 spectrophotometer (Düsseldorf, Germany). After each run, the photocatalytic membrane was cleaned by rinsing with distilled water containing 30% hydrogen peroxide under UV light for at least 2 h to restore catalytic activity.

### 2.3. Analysis of Photocatalytic Intermediates

Formation of intermediate products was investigated by GC–MS analysis (Agilent 6890 GC coupled to Agilent 5973 N MS, Santa Clara, CA, USA). Separation was performed on a HP-5MS column 30 m × 0.32 mm i.d., 25 µm film thickness (Santa Clara, CA, USA). The oven program started at 80 °C (3 min hold), followed by a ramp of 15 °C/min to 370 °C, maintained for 7 min. Diethyl ether was employed for extraction, with a solution-to-solvent ratio of 5:1. Single-step extractions were performed using anhydrous sodium sulfate to remove residual water.

### 2.4. Analysis of H_2_O_2_

Standard solutions (40 mmol/L) were prepared by diluting 30% (*v*/*v*) H_2_O_2_ with phosphate buffer (pH = 7.4) to a final volume of 50 mL [24]. Solution concentrations were verified based on a molar extinction coefficient of 43.6 M^−1^ · cm^−1^ at 240 nm. UV measurements were performed using a Shimadzu Spectrophotometer, model UV-1900 (Shimadzu Corporation, Kyoto, Japan) equipped with 1 cm quartz cells.

## 3. Results and Discussion

The photocatalytic degradation of 4-CP and 2,4-DCP was investigated at various initial concentrations, expressed as mg of organic carbon per liter (mg C L^−1^). For both substrates, the temporal evolution of the normalized concentration (C/C_0_) under UV irradiation clearly demonstrates a progressive decrease with increasing exposure time, confirming efficient photocatalytic oxidation leading toward mineralization.

**Figure 3:**
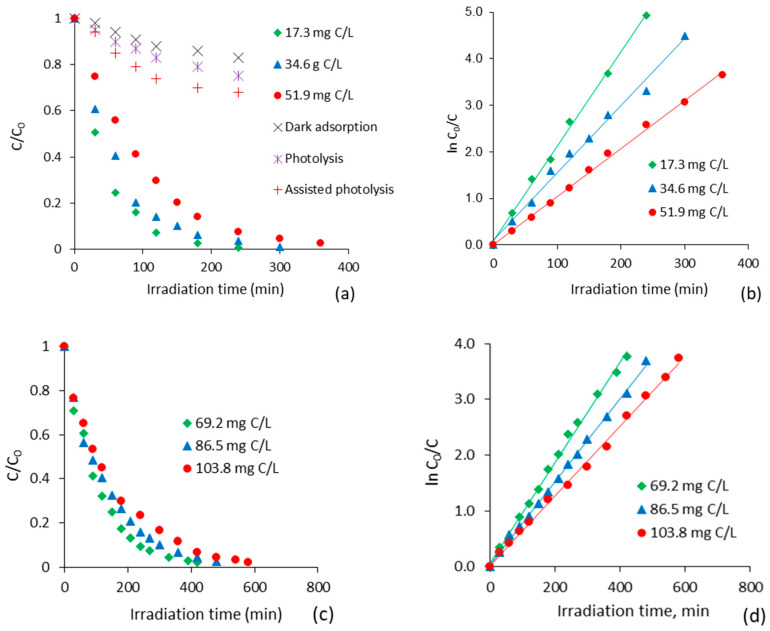
Photocatalytic degradation of 4-CP at different initial concentrations: (**a**,**c**) normalized concentration (C/C_0_) vs. irradiation time; (**b**,**d**) pseudo-first-order kinetic plots (ln(C_0_/C) vs. time). The results corresponding to dark adsorption, photolysis, and assisted photolysis (with addition of H_2_O_2_) are for the initial organic substrate solution of 17.3 mg C/L.

In the case of 4-CP, Figure 3a,c illustrate the decline in C/C_0_ with irradiation time for the tested concentrations (17.3–103.8 mg C L^−1^). The corresponding plots of ln(C_0_/C) versus irradiation time (Figure 3b,d) display linear relationships, indicating that the degradation follows pseudo-first-order kinetics. The apparent rate constant (k) decreases systematically with increasing substrate concentration, from 0.0203 min^−1^ at 17.3 mg C L^−1^ to 0.0063 min^−1^ at 103.8 mg C L^−1^ (Table 1). This trend is consistent with typical photocatalytic behavior, where higher organic substrate loading partially saturate active sites on the photocatalyst surface and increase competition for hydroxyl radicals, thus reducing the apparent reaction rate [25,26,27].

**Figure 4:**
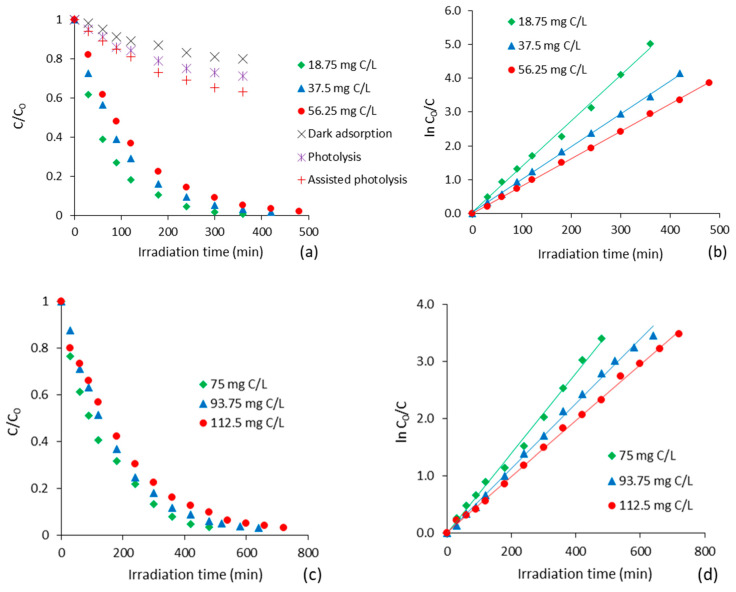
Photocatalytic degradation of 2,4-DCP at different initial concentrations: (**a**,**c**) normalized concentration (C/C_0_) vs. irradiation time; (**b**,**d**) pseudo-first-order kinetic plots (ln(C_0_/C) vs. time). The results corresponding to dark adsorption, photolysis, and assisted photolysis (with addition of H_2_O_2_) are for the initial organic substrate solution of 18.75 mg C/L.

For 2,4-DCP, a comparable degradation profile was observed (Figure 4). The C/C_0_ values decreased markedly over time for all tested concentrations (18.75–112.5 mg C L^−1^), confirming the photocatalytic system’s effectiveness. As with 4-CP, the ln(C_0_/C) versus time plots exhibited linear behavior at the lower concentrations, consistent with pseudo-first-order kinetics (Figure 4b,d). The apparent rate constant (*k*) decreased from 0.0203 min^−1^ at 18.75 mg C L^−1^ to 0.0063 min^−1^ at 112.5 mg C^−1^ (Table 1), again reflecting a concentration-dependent decline in reactivity.

Regarding the control experiments, only a slight degradation of the organic substrate was observed, whereas a pronounced improvement in degradation efficiency was achieved in the presence of the photocatalytic membrane, highlighting its key role in the process.

These kinetic observations should also be considered in relation to the reaction environment. In acidic conditions (pH 3), the surface of TiO_2_ (titanium dioxide) is expected to be positively charged, as the applied pH is below its point of zero charge (pH_pzc_, typically ~5–6). Under these conditions, the surface charge state may influence the adsorption behavior of reactants and intermediates, although electrostatic effects are partially mitigated by the speciation of the pollutants.

Indeed, both 4-CP and 2,4-DCP exhibit pH-dependent acid–base equilibria. At pH 3, which is below their pK_a_ (acid dissociation constant) values, these chlorophenolic compounds predominantly exist in their protonated (neutral) forms. Consequently, electrostatic interactions are not expected to play a dominant role in the adsorption process. Instead, pollutant removal is mainly governed by hydrogen bonding, hydrophobic interactions, and surface-mediated reactions driven by photogenerated reactive oxygen species.

**Figure 5:**
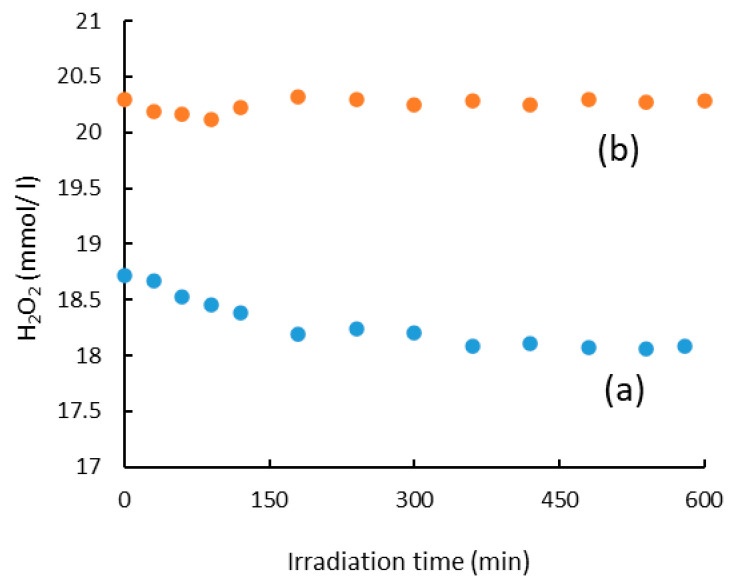
Evolution of H_2_O_2_ concentration during the photocatalytic degradation of 4-CP and 2,4-DCP using the TiO_2_-based photocatalytic membrane: (**a**) 4-CP; (**b**) 2,4-DCP.

Under UV irradiation, TiO_2_ generates reactive oxygen species, particularly hydroxyl radicals (•OH), which are responsible for the photocatalytic degradation of 4-CP and 2,4-DCP. Although H_2_O_2_ may additionally contribute to •OH formation, the experimental monitoring revealed only minor variations in H_2_O_2_ concentration throughout the process (Figure 5). These results indicate that H_2_O_2_ consumption remained limited relative to the initial oxidant dose, supporting the assumption of quasi-constant H_2_O_2_ concentration for kinetic interpretation.

To gain further insight into the degradation mechanism, the kinetic data were analyzed using the Langmuir–Hinshelwood (L–H) model, which describes surface-mediated reactions involving the adsorption of reactant molecules followed by surface oxidation. The general L–H rate expression is given by:

(1) $$r=\frac{k\;K\;C}{1+K\;C}$$

where *r* is the degradation rate (mg C·L^−1^·min^−1^), *k* is the intrinsic surface reaction rate constant (mg C L^−1^ min^−1^), *K* is the adsorption equilibrium constant (L mg C^−1^), and *C* is the instantaneous substrate concentration in solution (mg C L^−1^). The assumptions of this model include: (a) reversible adsorption of substrate on the catalyst surface according to a Langmuir isotherm, (b) the surface reaction between adsorbed substrate and reactive species (e.g., •OH) as the rate-determining step, and (c) rapid desorption of oxidation products such that adsorption equilibrium is maintained [28]. According to the model, two limiting kinetic conditions can be distinguished depending on the magnitude of the product KC:

*KC* ≪ 1 ⇒ *r* ≈ *kKC* = *k*_app_*C* (pseudo-first-order regime)

*KC* ≫ 1 ⇒ *r* ≈ *k* (zero-order regime: surface saturation)

Thus, when the concentration of the organic substrate is sufficiently low such that KC≪1, the denominator of Equation (1) approaches unity and the rate become directly proportional to the substrate concentration (i.e., r∝C). Under these conditions, the reaction follows an apparent first-order kinetic law, which explains the linear *ln(C*_0_*/C)*-versus-time plots observed experimentally. By contrast, at higher substrate concentrations, where KC becomes significant, the surface sites are increasingly occupied, and the rate approaches a limiting constant value independent of concentration (zero-order behavior). This limit behaviour in photocatalysis has been emphasized in recent kinetic reviews [29].

**Figure 6:**
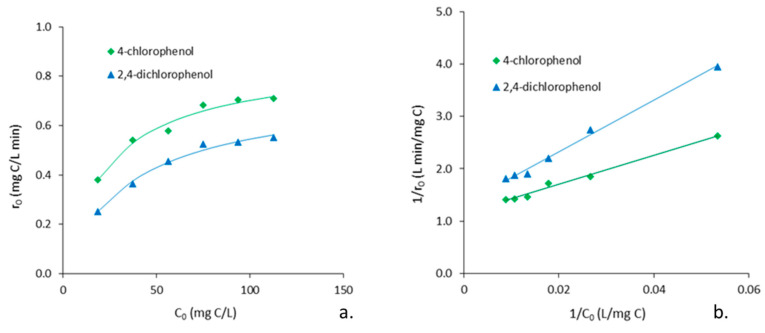
(**a**) Variation in the initial photocatalytic degradation rate ($r_{0}$) of 4-CP and 2,4-DCP as a function of the initial concentration ($C_{0}$), and (**b**) corresponding linearized L–H plots (1/$r_{0}$ versus 1/$C_{0}$).

The variation in the initial photocatalytic degradation rate (*r*_0_) with the initial substrate concentration (*C*_0_) for both 4-CP and 2,4-DCP is presented in Figure 6a, while the corresponding linearized L–H plots (1/*r*_0_ versus 1/*C*_0_) are shown in Figure 6b. The experimental data exhibit the characteristic saturation-type dependence expected for surface-mediated photocatalytic reactions, confirming that the process follows the L–H kinetic model (including the low-concentration first-order regime and high-concentration saturation regime) as discussed in recent studies [30,31,32]. As illustrated in Figure 6a, *r*_0_ increases with *C*_0_ for both compounds but gradually approaches a plateau at higher concentrations. This behavior reflects the transition from the adsorption-controlled, pseudo-first-order regime ($KC_{0}\ll 1$) to the surface-reaction-limited, zero-order regime ($KC_{0}\gg 1$), in accordance with Equation (2). The linear representation of the model:

(2) $$\frac{1}{r_{0}}=\frac{1}{k\;K}\cdot \frac{1}{C_{0}}+\frac{1}{k}$$

yields straight lines for both compounds, from which constants *k* and *K* were extracted (Table 1). The smaller slope obtained for 4-CP indicates a higher intrinsic rate constant and stronger adsorption affinity compared to 2,4-DCP [32].

The experimental data for 4-CP fit the L–H model well, yielding a surface reaction rate constant *k* = 0.872 mg C·L^−1^ min^−1^ and an adsorption constant *K* = 0.041 L·mg^−1^ C (Table 1), indicating moderate adsorption affinity for the photocatalyst surface. At lower concentrations (17.3–51.9 mg C L^−1^) nearly complete mineralization was achieved within approximately 300 min, whereas higher concentrations (≥69.2 mg C L^−1^) required up to 600 min for comparable degradation efficiency. In the case of 2,4-DCF, the L–H parameters obtained were *k* = 0.804 mg C·(L^−1^ min^−1^) and *K* = 0.024 L·mg^−1^ C, slightly lower than those for 4-CP, which suggests weaker adsorption on the photocatalyst surface.

Across the studied concentration range (0–120 mg C L^−1^), both phenolic compounds exhibit pseudo-first-order kinetics at low *C*_0_ values and gradually deviate toward zero-order behavior at higher loadings, consistent with the L–H mechanism and partial surface saturation. The maximum initial rate achieved for 4-CP (~0.8 mg C·L^−1^·min^−1^) exceeds that of 2,4-DCP (~0.6 mg C·L^−1^·min^−1^), confirming its greater photocatalytic reactivity under identical conditions. The observed differences in kinetic parameters and overall reactivity can be attributed to molecular structure. The presence of an additional chlorine substituent in 2,4-DCP increases molecular size and electron-withdrawing character, which reduces adsorption strength and decreases the accessibility of active sites for hydroxyl radical attack [33,34].

The GC–MS analysis provided in Table 2 reveals the formation of several key aromatic intermediates during the photocatalytic degradation of 4-CP and 2,4-DCP, allowing the elucidation of their transformation pathways under photocatalytic conditions. For 4-CP (RT = 6.28 min, *m*/*z* 65, 100, 128), the major detected products include hydroquinone (RT = 8.52 min), catechol (RT = 8.57 min), 4-chlorocatechol (RT = 10.14 min), and benzoquinone (RT = 4.17 min). The presence of both chlorinated (4-chlorocatechol) and dechlorinated (catechol, hydroquinone) intermediates indicates that the degradation proceeds through initial hydroxylation of the aromatic ring by photogenerated •OH radicals, followed by progressive dechlorination and oxidation steps, in agreement with reported degradation mechanisms for chlorinated phenols. The detection of benzoquinone, a typical oxidation product of phenolic species, further suggests that ring-opening reactions occur in later stages of the process [35,36].

Similarly, the degradation of 2,4-DCP (RT = 6.08 min, *m*/*z* 63, 162, 164) proceeds through a sequence of hydroxylation and dechlorination reactions. GC–MS identifies the formation of 2-chlorohydroquinone (RT = 10.74 min), 3,5-dichlorocatechol (RT = 12.48 min), 4-chlorophenol (RT = 6.28 min), and non-chlorinated species such as hydroquinone, catechol, and benzoquinone. The detection of 4-chlorophenol as an intermediate confirms that stepwise dechlorination occurs, likely promoted by electrophilic attack of hydroxyl radicals on the aromatic ring. The subsequent appearance of mono- and di-hydroxylated compounds (chlorinated or not) supports a degradation mechanism dominated by successive hydroxylation, dechlorination, and oxidation steps, features widely described in photocatalytic degradation pathways of chlorophenols. Therefore, the combination of characteristic retention times and diagnostic ions (*m*/*z*) demonstrates that both chlorophenolic substrates follow similar photocatalytic degradation routes, involving hydroxyl radical-mediated substitution of chlorine atoms, aromatic ring oxidation, and formation of quinone-type intermediates. These intermediates are consistent with commonly reported photocatalytic transformation pathways of chlorinated aromatic pollutants [37,38].

Peak-area integration of their specific ions was used as a semi-quantitative measure of relative concentration, allowing reconstruction of concentration–time profiles in the absence of full calibration curves. Therefore, this approach was used as a semi-quantitative indicator of the relative temporal evolution of the detected intermediates. These profiles served as the experimental basis for constructing and qualitatively validating the mechanistic kinetic model of the photocatalytic degradation pathways. The identified intermediates (Table 2) were proposed based on GC–MS library matching, retention behavior, and consistency with previously reported photocatalytic degradation mechanisms of chlorophenols. Additional confirmation using authentic standards or complementary analytical techniques (e.g., LC–MS/MS or high-resolution MS) would be necessary for definitive positional isomer identification and will be considered in future studies.

The photocatalytic transformation of both 4-CP and 2,4-DCP observed by GC–MS (Table 2) can be described by a set of coupled L–H-type non-linear kinetic equations that explicitly account for competitive adsorption of both the parent substrate and the individual intermediates on the active sites of the photocatalyst (adsorption–reaction sequence). In the expanded kinetic formulation used here, the time evolution of the concentration of the substrate and of the main ring-oxidation intermediates detected by GC–MS is governed by first-order differential non-linear equations with Langmuir denominators that express mutual competition for surface sites. This expanded model is described by the equations presented below (Table 3 and Table 4).

In these expressions *C*_4-*CP*_, *C*_2,4-*DCP*_, *C_HQ_*, *C*_2-*CHQ*_, *C*_3,5-*DCC*_, *C*_4-*CC*_, *C_BQ_*, and *C_C_* are the bulk concentrations (mg·L^−1^) of both initial organic substrates (4-CP and 2,4-DCP) and organic intermediates that are formed during the irradiation period (Table 2). The kinetic expressions include multiple additive terms in the numerator to account for parallel reaction pathways occurring on the catalyst surface. Each term represents an independent Langmuir–Hinshelwood surface reaction involving the adsorbed parent compound, leading to different intermediates. The overall reaction rate is therefore expressed as the sum of these competitive pathways. Therefore, the differences in the Langmuir denominator terms arise from the competitive adsorption of specific reactants and intermediates involved in each elementary step. Although multiple species are present in the reaction medium, each kinetic expression considers only those compounds that actively compete for adsorption on the catalyst surface in that particular reaction pathway, according to the Langmuir–Hinshelwood formalism.

Hydrogen peroxide (H_2_O_2_) was used in a fixed initial stoichiometric amount and was not considered a dynamic species in the kinetic model. Under UV irradiation, H_2_O_2_ primarily acts as an additional source of hydroxyl radicals (•OH), and its effect is therefore included implicitly within the apparent rate constants of the Langmuir–Hinshelwood kinetic formulation.

The parameters $k_{i}$ are the apparent rate constants (reaction constant per adsorbed species) and $K_{i}$ are the Langmuir apparent adsorption equilibrium constants (site affinity) for the corresponding adsorption–reaction steps. Numerators in each rate expression represent productive formation and/or consumption terms (surface reaction rates scaled by adsorption), while the denominators $\left(1+\sum K_{j}C_{j}\right)$ implement competitive adsorption among species, reducing the effective reaction rate as sites become occupied, a hallmark of the L–H approach to heterogeneous photocatalysis.

Practically, the expanded systems (Equations (3)–(7) for 4-CP and Equations (8)–(13) for 2,4-DCP) must be integrated simultaneously because the production of each intermediate depends on the instantaneous concentrations of precursor species and on competitive site occupancy. Although reduced formulations exist that lump intermediates into a single “intermediate” pool [9], the present sets explicitly resolve the main GC–MS identified intermediates (Table 2), which improves mechanistic interpretation and allows direct comparison between simulated and measured concentrations of individual species.

The apparent kinetic parameters for the photocatalytic degradation of both 4-CP and 2,4-DCP were obtained through a structured procedure. The primary parameters $k_{1}$ and $K_{1}$, governing the initial degradation step, were estimated from the linearized L–H model using short-time experimental rates, where intermediate species are negligible. These values (Table 1) provided physically meaningful starting points for the full nonlinear kinetic model. The remaining kinetic and adsorption constants ($k_{2},K_{2},k_{3},K_{3},\ldots$) were subsequently determined by sequential fitting, adjusting each parameter pair according to the formation and consumption of the corresponding intermediate.

Finally, a global optimization was performed using all experimental concentration–time datasets simultaneously, by minimizing the weighted sum-of-squares objective function:

(14) $$F(p)=\sum_{i=1}^{n}w_{i}{\left(C_{i}^{exp}-C_{i}^{calc}(p)\right)}^{2}$$

where *p* is the vector of all kinetic parameters, $C_{i}^{exp}$ and $C_{i}^{calc}$ are the measured and model-predicted concentrations of the substrate or intermediates at time point i, and the weight $w_{i}=1/\sigma^{2}\left(C_{i}^{exp}\right)$ accounts for heteroscedastic measurement uncertainty. This global optimization yields a robust and consistent parameter set that accurately reproduces the observed concentration profiles for both the substrate and its intermediates across all experiments.

Numerical solutions of the system and parameter optimization were performed by (a) forward integration of the coupled Ordinary Differential Equations (ODEs) (Equations (3)–(7) for 4-CP and Equations (8)–(13) for 2,4-DCP) for a trial parameter vector to obtain $C_{i}^{\mathrm{calc}}$ at the experimental time points (Runge–Kutta method), and (b) application of a nonlinear optimization algorithm (Levenberg–Marquardt) to minimize F(p). Confidence intervals for the fitted parameters were estimated either from the covariance matrix of the fit or by bootstrap/resampling procedures, which also help assess parameter identifiability—an important aspect given that competitive adsorption often introduces strong correlations between $k_{i}$ and $K_{i}$.

**Figure 7:**
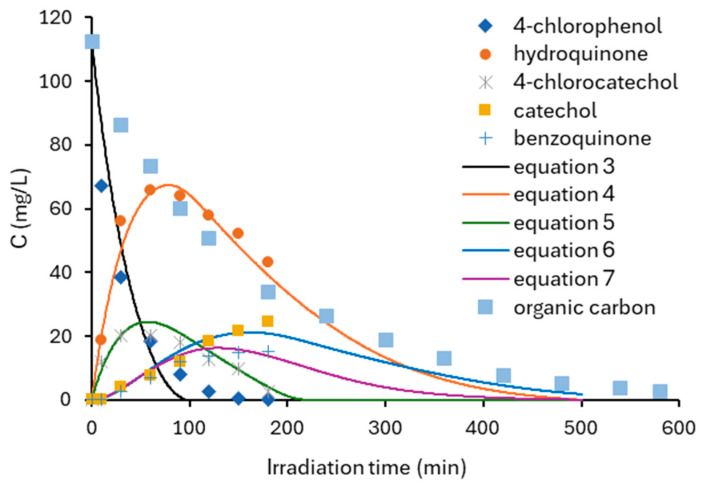
Concentration–time profiles of 4-CP and its aromatic intermediates: experimental data and nonlinear kinetic model predictions.

The concentration–time profiles of 4-CP and its main aromatic intermediates are presented in Figure 7. The resulting model provides an excellent fit for experimental observations, capturing both the formation and decay of intermediates such as hydroquinone, 4-chlorocatechol, catechol, and benzoquinone. The corresponding apparent kinetic constants obtained from the nonlinear regression analysis are summarized in Table 5. The parent compound, 4-chlorophenol, is characterized by two kinetic stages: an initial rapid degradation step (*k*_1_ = 0.290 mg·min^−1^, *K*_1_ = 0.073 mg^−1^) followed by a slower transformation (*k*_2_ = 0.111 mg·min^−1^, *K*_2_ = 0.091 mg^−1^), indicating that both direct photocatalytic oxidation and subsequent hydroxylation reactions contribute to its removal. Among the intermediates, hydroquinone exhibits a relatively high-rate constant (*k*_3_ = 0.621 mg·min^−1^), suggesting that it is rapidly formed and consumed within the reaction network. Similarly, 4-chlorocatechol (*k*_4_ = 0.401 mg·min^−1^) and catechol (*k*_5_ = 0.783 mg·min^−1^) are transient species that undergo further oxidation, while benzoquinone shows the highest degradation rate (*k*_6_ = 0.984 mg·min^−1^) and moderate adsorption affinity (*K*_5_ = 0.065 mg^−1^), confirming its role as a short-lived intermediate prior to mineralization.

The nonlinear kinetic simulation accurately reproduces the temporal profiles of each compound, validating the proposed mechanism. The model also predicts the gradual decrease in total organic carbon (TOC), consistent with the experimental measurements and confirming that complete mineralization occurs after approximately 500–600 min of irradiation. Gas chromatography analysis supports the modeling results, verifying the presence and sequential disappearance of the predicted intermediates.

Both the GC-MS and kinetic model suggest two simultaneous degradation pathways for 4-CP, namely:

*Pathway I: Hydroquinone route*

Hydroquinone is formed through hydroxylation and partial dechlorination:

(15) $$4\text{-}CP+•OH\to HQ+Cl^{-}$$

Hydroquinone is subsequently oxidized to p-benzoquinone:

(16) $$HQ+•OH\to BQ+2H^{+}+2e^{-}$$

Benzoquinone then undergoes further oxidative degradation and aromatic ring opening:

(17) $$BQ+•OH\to \mathrm{organic}\;\mathrm{acids}\to CO_{2}+H_{2}O$$

*Pathway II: Chlorocatechol route*

4-chlorocatechol is generated by hydroxyl radical attack on the aromatic ring:

(18) 4-CP+•OH→4-CC

The chlorinated intermediate is further converted into catechol through oxidative dechlorination:

(19) $$4\text{-}CC+•OH\to C+Cl^{-}$$

Catechol is subsequently oxidized into low-molecular-weight intermediates and finally mineralized:

(20) $$C+•OH\to \mathrm{organic}\;\mathrm{acids}\to CO_{2}+H_{2}O$$

**Figure 8:**
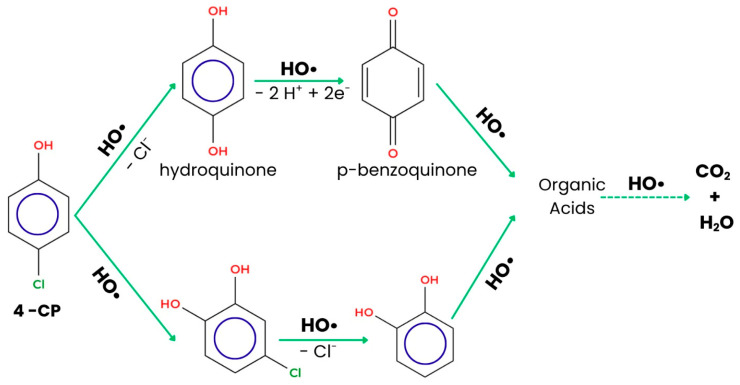
Proposed photocatalytic degradation pathway of 4-CP over stainless-steel/TiO_2_ membrane under UV irradiation.

A schematic representation of the proposed photocatalytic degradation mechanism of 4-CP based on the detected intermediates and kinetic modeling results is presented in Figure 8.

**Figure 9:**
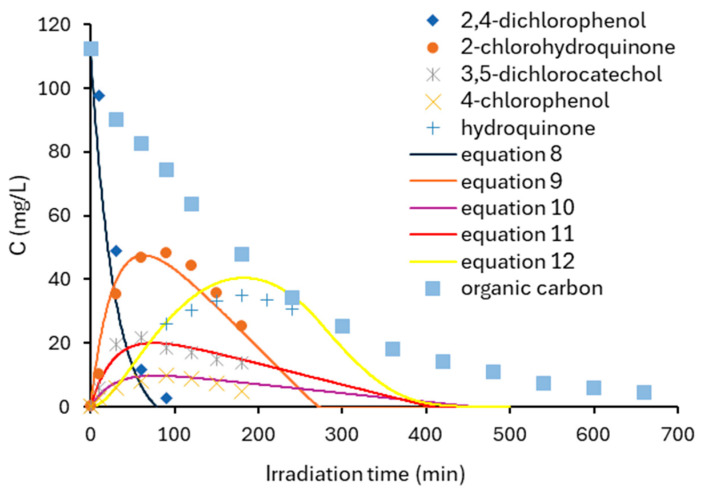
Concentration–time profiles of 2,4-DCP and its aromatic intermediates: experimental data and nonlinear kinetic model predictions.

The concentration–time profiles of 2,4-DCP and its transformation intermediates are illustrated in Figure 9. The model closely reproduces the experimental data, confirming the applicability of the nonlinear kinetic approach to describe the complex photocatalytic pathway. At the initial stage of irradiation, the concentration of 2,4-DCP decreases sharply, indicating rapid adsorption on the catalyst surface and efficient photocatalytic oxidation. The degradation proceeds through a multistep mechanism involving the formation of several intermediates, including 2-chlorohydroquinone, 3,5-dichlorocatechol, 4-chlorophenol, hydroquinone, and 4-chlorocatechol. The temporal evolution of these species reveals that 2-chlorohydroquinone and 3,5-dichlorocatechol are the predominant intermediates during the early stages, while 4-chlorophenol and hydroquinone appear later in the reaction, suggesting successive dechlorination and hydroxylation steps.

The apparent kinetic parameters obtained from nonlinear fitting are summarized in Table 6. The parent compound 2,4-dichlorophenol exhibits three distinct degradation stages, with apparent rate constants *k*_1_ = 0.210 mg·min^−1^ (*K*_1_ = 0.095 mg^−1^), *k*_2_ = 0.250 mg·min^−1^ (*K*_2_ = 0.017 mg^−1^), and *k*_3_ = 0.411 mg·min^−1^ (*K*_3_ = 0.020 mg^−1^), indicating an initially slower process that accelerates as the molecule undergoes dechlorination. The intermediate 2-chlorohydroquinone shows moderate reactivity (*k*_4_ = 0.110 mg·min^−1^), while 3,5-dichlorocatechol displays a higher rate (*k*_5_ = 0.216 mg·min^−1^), consistent with its faster conversion. The formation of 4-chlorophenol through consecutive steps (*k*_6_ = 0.112, *k*_7_ = 0.295, *k*_8_ = 0.116 mg·min^−1^) confirms that partial dechlorination is a key route in the overall process. Hydroquinone exhibits the highest degradation rate (*k*_9_ = 0.869 mg·min^−1^), indicating rapid oxidation into benzoquinone and subsequently into CO_2_ and H_2_O. Finally, 4-chlorocatechol (*k*_10_ = 0.315 mg·min^−1^, *K*_10_ = 0.076 mg^−1^) appears as a short-lived intermediate before complete mineralization.

The concentration profiles of the detected intermediates for both 4-CP and 2,4-DCP were estimated from GC–MS chromatographic peak integration and should therefore be considered semi-quantitative. The obtained kinetic parameters correspond to apparent nonlinear fitting constants associated with the proposed Langmuir–Hinshelwood reaction network. Although the experimental degradation trends showed satisfactory reproducibility, a detailed statistical confidence interval analysis of the fitted parameters was beyond the scope of the present work.

The nonlinear kinetic simulation shows a high degree of correlation with the experimental results, reproducing both the transient accumulation of intermediates and the overall decrease in total organic carbon (TOC). The progressive decline in TOC confirms that the reaction proceeds toward complete mineralization over approximately 600 min of irradiation. Gas chromatography analyses confirmed the presence of the same intermediates predicted by the model, validating the proposed reaction sequence.

Nonlinear modeling offers significant advantages over classical L–H approaches in studying the photocatalytic degradation of chlorinated phenols. Unlike the standard L–H model, which simplifies kinetics based on substrate adsorption and surface reaction, nonlinear modeling explicitly accounts for the complex interactions between the parent compound, intermediates, and reactive species. This approach captures the multi-step kinetics, including the transient formation and consumption of intermediates such as hydroquinone, catechol, and chlorocatechols, which cannot be fully described by linearized L–H models. Furthermore, nonlinear modeling incorporates competitive adsorption on catalyst sites and dynamic concentration changes over time, providing a more accurate representation of the actual reaction mechanism. As a result, it not only reproduces the experimental concentration–time profiles with high fidelity but also enables the quantitative estimation of kinetic parameters for individual steps, supporting mechanistic interpretation and optimization of photocatalytic processes.

Both the GC-MS and kinetic model suggest four simultaneous degradation pathways for 2,4-DCP, namely:

*Pathway I: Hydroquinone route*

Hydroquinone is formed through hydroxylation and partial dechlorination:

2,4-DCF + •OH → HQ + Cl^−^ (21)

Hydroquinone is subsequently oxidized to p-benzoquinone:

HQ + •OH → BQ + 2H^+^ + 2e^−^ (22)

Benzoquinone then undergoes further oxidative degradation and aromatic ring opening:

BQ + •OH → organic acids → CO_2_ + H_2_O (23)

*Pathway II: Chlorohydroquinone route*

2-chlorohydroquinone is generated by hydroxyl radical attack on the aromatic ring:

2,4-DCF + •OH → 2-CHQ(24)

The chlorinated intermediate is further converted into hydroquinone through oxidative dechlorination:

2-CHQ + •OH → HQ + Cl^−^ (25)

Hydroquinone is subsequently oxidized into low-molecular-weight intermediates and finally mineralized:

HQ + •OH → organic acids → CO_2_ + H_2_O (26)

*Pathway III: Chlorocatechol route*

3,5-dichlorocatechol is generated by hydroxyl radical attack on the aromatic ring:

2,4-DCF + •OH → 3,5-DCC(27)

The chlorinated intermediate is further converted into chlorocatechol through oxidative dechlorination:

3,5-DCC + •OH → 4-CC + Cl^−^ (28)

Chlorocatechol is subsequently oxidized into low-molecular-weight intermediates and finally mineralized:

4-CC + •OH → organic acids → CO_2_ + H_2_O (29)

*Pathway IV: 4-chlorophenol route*

4-chlorophenol is formed through hydroxyl radical transformation of the parent compound:

2,4-DCF + •OH → 4-CP(30)

4-chlorophenol follows two competitive degradation routes:

4-CP + •OH → HQ + Cl^−^ (31)

4-CP + •OH → 4-CC(32)

Both intermediates are further oxidized into low-molecular-weight compounds and finally mineralized:

HQ/4-CC + •OH → organic acids → CO_2_ + H_2_O (33)

**Figure 10:**
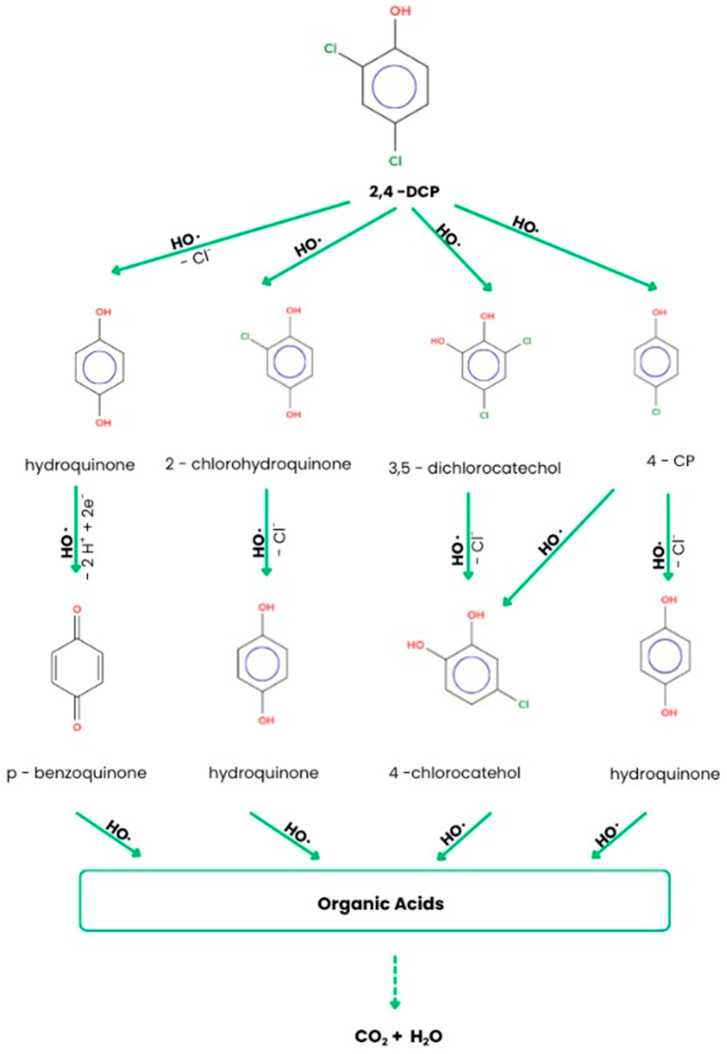
Proposed photocatalytic degradation pathway of 2,4-DCP over stainless-steel/TiO_2_ membrane under UV irradiation.

A schematic representation of the proposed photocatalytic degradation mechanism of 2,4-DCP based on the detected intermediates and kinetic modeling results is presented in Figure 10.

The proposed photocatalytic degradation pathways and the involvement of reactive oxygen species were inferred from the identified degradation intermediates, kinetic modeling results, and previously reported mechanisms for TiO_2_/H_2_O_2_/UV systems. Since no direct radical identification experiments (e.g., EPR spectroscopy or radical scavenger tests) were performed in the present study, the participation of •OH radicals and related reactive species should be regarded as mechanistically supported but indirectly evidenced. Further investigations involving direct radical detection and catalyst defect analysis will be considered in future work.

Overall, the strong correlation between experimental and simulated data demonstrates that the nonlinear kinetic model provides a robust and predictive description of the photocatalytic degradation of both 4-CP and 2,4-DCP. This modeling approach not only elucidates the transformation pathway but also allows for the quantitative evaluation of individual reaction steps, contributing to a deeper understanding of photocatalytic mechanisms relevant to environmental remediation.

Although simplified kinetic approaches may provide satisfactory fitting for the degradation of the parent compound, they cannot adequately describe the simultaneous formation and consumption of intermediate species observed experimentally. Therefore, a nonlinear Langmuir–Hinshelwood model incorporating parallel reaction pathways and competitive adsorption effects was adopted to better represent the mechanistic complexity of the photocatalytic system. Further validation of the proposed model using additional photocatalytic materials represents an important direction for future studies.

## 4. Conclusions

This study proposes a nonlinear mechanistic and kinetic framework for chlorophenol photocatalytic degradation by combining experimentally identified intermediates with nonlinear Langmuir–Hinshelwood modeling. The developed nonlinear model incorporates competitive adsorption, parallel degradation pathways, and intermediate-specific reaction steps, providing a more comprehensive description of the photocatalytic oxidation mechanism for both 4-chlorophenol and 2,4-dichlorophenol systems. Photocatalytic oxidation using the TiO_2_-based membrane efficiently degraded 4-CP and 2,4-DCP, achieving near-complete mineralization within 500–600 min under UV irradiation. The formation of CO_2_ and complete mineralization were inferred from the proposed photocatalytic degradation pathways and previously reported mechanisms for TiO_2_-based systems. Degradation followed pseudo-first-order kinetics at low concentrations and gradually transitioned to a zero-order regime at higher loadings, consistent with L–H surface saturation effects. GC–MS analysis confirmed the formation of key intermediates, including hydroquinone, catechol, chlorocatechols, and benzoquinone, revealing multi-step hydroxylation, dechlorination, and oxidation pathways. By implementing nonlinear kinetic modeling of coupled differential equations, the study captured the dynamic evolution of both substrates and intermediates, including transient accumulation and consumption patterns that classical linearized L–H models cannot adequately describe. This approach allowed estimation of kinetic and adsorption parameters for each reaction step, reflecting the relative reactivity and surface affinity of the species involved. The close agreement between experimental and simulated concentration–time profiles validate the proposed multi-step mechanism and demonstrates the predictive power of nonlinear modeling, offering a reliable framework for mechanistic interpretation and optimization of photocatalytic treatment strategies for chlorinated phenols.

## Figures and Tables

**Table 1:** Kinetic parameters for the photocatalytic degradation of 4-CP and 2,4-DCP.

Initial Organic Substrate Concentration C_0_ (mg C L^−1^)	Pseudo-First-Order Rate Constant k (min^−1^)	Initial Rate r_0_ (mg C L^−1^ min^−1^)	k (L−H), (mg C L^−1^ min^−1^)	K (L−H), (L mg^−1^ C)
*4-CP*				
17.30 (50 mg O_2_/L)	0.0203	0.381	0.872	0.041
34.60 (100 mg O_2_/L)	0.0144	0.540		
51.90 (150 mg O_2_/L)	0.0103	0.579		
69.20 (200 mg O_2_/L)	0.0091	0.683		
86.50 (250 mg O_2_/L)	0.0075	0.703		
103.80 (300 mg O_2_/L)	0.0063	0.709		
*2,4-DCP*				
18.75 (50 mg O_2_/L)	0.0203	0.253	0.804	0.024
37.50 (100 mg O_2_/L)	0.0144	0.364		
56.25 (150 mg O_2_/L)	0.0103	0.456		
75.00 (200 mg O_2_/L)	0.0091	0.525		
93.75 (250 mg O_2_/L)	0.0075	0.534		
112.50 (300 mg O_2_/L)	0.0063	0.551		

**Table 2:** Retention time (RT) and selected characteristic ions (*m*/*z*) for the identified organic compounds.

Organic Compound	RT (min)	*m*/*z*
*Initial organic substrate: 4-CP*		
4-chlorophenol	6.28	65, 100, 128
hydroquinone	8.52	63, 81, 110
4-chlorocatechol	10.14	63, 98, 144
catechol	8.57	64, 109, 110
benzoquinone	4.17	54, 82, 108
*Initial organic substrate: 2,4-DCP*		
2,4-dichlorophenol	6.08	63, 162, 164
2-chlorohydroquinone	10.74	52, 144, 146
3,5-dichlorocatechol	12.48	114, 178, 180
4-chlorophenol	6.28	65, 100, 128
hydroquinone	8.52	63, 81, 110
4-chlorocatechol	10.14	63, 98, 144
catechol	8.57	64, 109, 110

**Table 3:** The set of nonlinear differential equations that describes the kinetics of the photocatalytic degradation of 4-CP.

$-\frac{dC_{4-CP}}{dt}=\frac{k_{1}K_{1}C_{\left(4-CF\right)}+k_{2}K_{2}C_{\left(4-CF\right)}}{1+K_{1}C_{\left(4-CP\right)}+K_{2}C_{\left(4-CP\right)}}$	(3)
$\frac{dC_{HQ}}{dt}=\frac{k_{1}K_{1}C_{\left(4-CF\right)}-k_{3}K_{3}C_{\left(HQ\right)}}{1+K_{1}C_{\left(4-CF\right)}+K_{3}C_{\left(HQ\right)}}$	(4)
$\frac{dC_{4-CC}}{dt}=\frac{k_{2}K_{2}C_{\left(4-CF\right)}-k_{4}K_{4}C_{\left(4-CC\right)}}{1+K_{2}C_{\left(4-CF\right)}+K_{4}C_{\left(4-CC\right)}}$	(5)
$\frac{dC_{BQ}}{dt}=\frac{k_{3}K_{3}C_{\left(HQ\right)}-k_{5}K_{5}C_{\left(BQ\right)}}{1+K_{3}C_{\left(HQ\right)}+K_{5}C_{\left(BQ\right)}}$	(6)
$\frac{dC_{C}}{dt}=\frac{k_{4}K_{4}C_{\left(4-CC\right)}-k_{6}K_{6}C_{\left(C\right)}}{1+K_{4}C_{\left(4-CC\right)}+K_{6}C_{\left(C\right)}}$	(7)

**Table 4:** The set of nonlinear differential equations that describes the kinetics of the photocatalytic degradation of 2,4-CP.

$\frac{dC_{2,4-2CP}}{dt}=\frac{k_{1}K_{1}C_{\left(2,4-DCF\right)}+k_{2}K_{2}C_{\left(2,4-DCF\right)}+k_{3}K_{3}C_{\left(2,4-DCF\right)}}{1+K_{1}C_{\left(2,4-2CP\right)}+K_{2}C_{\left(2,4-DCP\right)}+K_{3}C_{\left(2,4-DCP\right)}}$	(8)
$\frac{dC_{2-CHQ}}{dt}=\frac{k_{1}K_{1}C_{\left(2,4-DCF\right)}-k_{4}K_{4}C_{\left(2-CHQ\right)}}{1+K_{1}C_{\left(2,4-DCF\right)}+K_{4}C_{\left(2-CHQ\right)}}$	(9)
$\frac{dC_{3,5-DCC}}{dt}=\frac{k_{2}K_{2}C_{\left(2,4-DCF\right)}-k_{5}K_{5}C_{\left(3,5-DCC\right)}}{1+K_{2}C_{\left(2,4-DCF\right)}+K_{5}C_{\left(3,5-DCC\right)}}$	(10)
$\frac{dC_{4-CP}}{dt}=\frac{k_{3}K_{3}C_{\left(2,4-DCF\right)}-k_{6}K_{6}C_{\left(4-CP\right)}}{1+K_{3}C_{\left(2,4-DCF\right)}+K_{6}C_{\left(4-CP\right)}}$	(11)
$\frac{dC_{HQ}}{dt}=\frac{k_{4}K_{4}C_{\left(2-CHQ\right)}+k_{7}K_{7}C_{\left(4-CP\right)}-k_{9}K_{9}C_{\left(HQ\right)}}{1+K_{4}C_{\left(2-CHQ\right)}+K_{7}C_{\left(4-CP\right)}+K_{9}C_{\left(HQ\right)}}$	(12)
$\frac{dC_{4-CC}}{dt}=\frac{k_{4}K_{4}C_{\left(3,5-DCC\right)}+k_{8}K_{8}C_{\left(4-CP\right)}-k_{10}K_{10}C_{\left(4-CC\right)}}{1+K_{4}C_{\left(3,5-DCC\right)}+K_{8}C_{\left(4-CP\right)}+K_{10}C_{\left(4-CC\right)}}$	(13)

**Table 5:** The apparent kinetic parameters for the degradation mechanism of 4-CP.

Organic Compounds	k (mg C L^−1^ min^−1^)		K (L mg^−1^ C)	
4-chlorophenol (4-CP)	k_1_	0.290	K_1_	0.073
	k_2_	0.111	K_2_	0.091
Hydroquinone (HQ)	k_3_	0.621	K_3_	0.011
4-chlorocatechol (4-CC)	k_4_	0.401	K_4_	0.082
Catechol (C)	k_5_	0.783	K_4_	0.013
Benzoquinone (BQ)	k_6_	0.984	K_5_	0.065

**Table 6:** The apparent kinetic parameters for the degradation mechanism of 2,4-DCP.

Organic Compounds	k (mg C L^−1^ min^−1^)		K (L mg^−1^ C)	
2,4-dichlorophenol (2,4-DCF)	k_1_	0.210	K_1_	0.095
	k_2_	0.250	K_2_	0.017
	k_3_	0.411	K_3_	0.020
2-chlorohydroquinone (2-CHQ)	k_4_	0.110	K_4_	0.052
3,5-dichlorocatechol (3,5-DCC)	k_5_	0.216	K_5_	0.021
4-chlorophenol (4-CP)	k_6_	0.112	K_6_	0.013
4-chlorophenol (4-CP)	k_7_	0.295	K_7_	0.015
	k_8_	0.116	K_8_	0.012
Hydroquinone (HQ)	k_9_	0.869	K_9_	0.027
4-chlorocatechol (4-CC)	k_10_	0.315	K_10_	0.076

## Data Availability

The data presented in this study are available on request from the corresponding author. Data are not publicly available due to privacy restrictions.
